# The Digital Information Environment of Lung Cancer and Lung Cancer Screening: Protocol for a Cross-Platform Social Media Content Analysis

**DOI:** 10.2196/89479

**Published:** 2026-03-30

**Authors:** Lisa Carter-Bawa, Ana Guadalupe Vielma, Gia Nealy, Diya Vemuganti, Nidhi Patel

**Affiliations:** 1 Cancer Prevention Precision Control Institute, Center for Discovery & Innovation, Hackensack Meridian Health, 123 Metro Blvd, 6th Floor, 6400 Pod, Nutley, NJ, 07110, United States, 1-201-880-3443

**Keywords:** lung cancer screening, low-dose computed tomography, social media, digital health, misinformation, stigma, nihilism, health communication, content analysis, implementation science

## Abstract

**Background:**

Lung cancer screening (LCS) with low-dose computed tomography reduces mortality by up to 20%, yet uptake in the United States remains below 6% of eligible individuals. Factors contributing to low uptake include lack of awareness, eligibility confusion, stigma associated with smoking history, and nihilistic beliefs about outcomes. Stigma triggers shame-avoidance behaviors, nihilism undermines perceived screening benefit, and misinformation amplifies both by spreading inaccurate eligibility criteria and exaggerated harms. Social media increasingly shapes how individuals encounter health information, form risk perceptions, and make screening decisions. Because platform architectures differ in content modality, algorithmic curation, and user demographics, single-platform studies cannot reliably characterize the digital information environment or identify platform-specific intervention targets.

**Objective:**

This study aims to (1) systematically characterize the clinical accuracy, stigma prevalence, and decision-support quality of lung cancer and screening content across 7 major social media platforms; (2) quantify platform-specific patterns in stigma manifestation and nihilistic messaging; (3) test whether inaccurate or stigmatizing content is associated with disproportionate engagement relative to accurate, nonstigmatizing content; and (4) as an exploratory aim, identify digital opinion leaders who could serve as partners for evidence-based dissemination.

**Methods:**

This cross-sectional content analysis will examine publicly accessible posts from Facebook, Instagram, TikTok, YouTube, X/Twitter, Reddit, and Bluesky. Posts will be identified through predefined search terms across 2 content domains: LCS and lung cancer narratives (diagnosis, treatment, survivorship). The sampling strategy combines relevance-based sampling, targeting approximately 700‐1000 unique posts after deduplication—a sample size providing 80% power for cross-platform comparisons assuming medium effect sizes. A structured codebook operationalizing constructs from diffusion of innovations theory, attribution theory of stigma, and health misinformation frameworks will assess accuracy, stigma, decision support, and equity. All posts will be dual-coded by trained coders. Interrater reliability will be assessed using Gwet’s AC1. Analyses will include descriptive statistics, cross-platform comparisons using chi-square and Kruskal-Wallis tests, and negative binomial regression models testing whether accuracy and stigma characteristics predict engagement.

**Results:**

Data collection began in October 2025 and is projected to be complete by July 2026. As of March 2026, data have been collected from 181 posts across 7 platforms. Results are expected to be published by December 2026. Findings will characterize accuracy patterns, stigma prevalence, benefit-harm framing, and engagement dynamics across platforms, informing clinical communication tools, navigator training, and digital intervention development.

**Conclusions:**

This protocol describes the first multiplatform, theory-informed analysis of lung cancer and LCS content on social media. The study will generate foundational evidence to inform stigma-informed communication strategies, decision support tools, and equitable dissemination approaches. The methodology provides a replicable framework for monitoring health information ecosystems across disease contexts.

## Introduction

### Background

Lung cancer remains the leading cause of cancer-related death in the United States, accounting for approximately 125,000 deaths annually [1]. Screening with low-dose computed tomography (LDCT) of the chest has been shown to reduce lung cancer mortality by 20% among high-risk individuals in the National Lung Screening Trial [2] and by 24% among men in the NELSON trial [3]. Based on this evidence, the United States Preventive Services Task Force (USPSTF) recommends annual LDCT screening for adults aged 50‐80 years with a 20 pack-year smoking history who currently smoke or quit within the past 15 years [4].

Despite this evidence, lung cancer screening (LCS) uptake remains alarmingly low. Recent estimates suggest that fewer than 6% of eligible individuals undergo screening annually [5], with substantial disparities by race, socioeconomic status, geography, and insurance coverage [6]. Multiple factors contribute to low uptake, including limited public awareness of screening availability, confusion about eligibility criteria, uncertainty about benefits and risks, logistical barriers, and clinician-level factors such as competing clinical priorities and insufficient time for shared decision-making [7,8].

Uniquely among cancer screenings, lung cancer carries substantial stigma rooted in its association with tobacco use. This stigma manifests as blame, shame, and social devaluation directed toward individuals with lung cancer or those at risk, regardless of their actual smoking history [9]. Stigma operates at multiple levels—internalized shame among patients, interpersonal blame from family and health care clinicians, and structural discrimination in resource allocation—and has been linked to delayed care-seeking, psychological distress, and poorer quality of life [10,11]. Closely related to stigma is therapeutic nihilism: the belief that lung cancer is uniformly fatal and that screening or treatment is futile [12]. Nihilistic beliefs may discourage both patients and clinicians from pursuing early detection, undermining the potential benefits of screening.

These constructs—stigma, nihilism, and misinformation—operate through distinct but interconnected pathways to undermine screening uptake. Stigma functions at the individual level by triggering shame-avoidance behaviors (avoiding screening to avoid confronting smoking history) and at the interpersonal level by discouraging disclosure and help-seeking [10]. Nihilism, the belief that lung cancer is uniformly fatal, undermines the perceived benefit of early detection, a core prerequisite for screening adoption under rational decision-making models [12]. Misinformation can amplify both constructs: inaccurate eligibility information excludes individuals who might benefit, while exaggerated harms or minimized benefits reinforce nihilistic beliefs. When these constructs converge in high-visibility social media content, they may create a self-reinforcing information ecosystem that systematically disadvantages informed screening decisions.

Social media has become a dominant channel through which individuals encounter health information, including cancer-related content [13]. Platforms such as YouTube, TikTok, Instagram, and Facebook host millions of health-related posts that shape public understanding of disease risk, screening options, and treatment outcomes [14]. For lung cancer specifically, social media serves as a space where survivors share narratives, clinicians disseminate guidance, advocacy organizations promote awareness, and misinformation circulates alongside evidence-based content. The algorithmic curation systems of these platforms may preferentially surface content that generates high engagement, potentially resulting in greater visibility for emotionally charged, sensational, or stigmatizing narratives relative to balanced, accurate information [15].

Despite the growing influence of social media on health perceptions and behaviors, the digital information environment for lung cancer and LCS has received limited systematic attention. Existing research has been confined to single-platform analyses [16,17], narrow constructs such as sentiment or misinformation [18], or cancer types other than lung cancer [19,20]. These limitations are consequential for 3 reasons. First, platform architectures differ fundamentally in content modality (video vs text), algorithmic curation, and user demographics, making cross-platform generalization from single-platform studies unreliable. Second, health information seekers increasingly navigate multiple platforms, encountering potentially contradictory messages that shape cumulative perceptions—a phenomenon invisible to single-platform research. Third, stigma and misinformation may manifest differently across platforms due to content format constraints and community norms. Short-form video platforms such as TikTok and Instagram Reels privilege emotional and narrative content—personal testimonials, fear appeals, and survivor stories—that may convey stigma through visual and affective cues rather than explicit claims. Text-based platforms such as Reddit and X/Twitter enable more detailed informational content, including eligibility discussions that may be accurate or misleading, and community norms that shape whether stigmatizing language is challenged or reinforced. These modality-driven differences are expected to shape both the nature of misinformation and the audience exposed to it, and they draw fundamentally different audiences with distinct information-processing patterns. No prior work has systematically compared clinical accuracy, stigma and nihilism, benefit-harm framing, representation and equity, and engagement dynamics across the dominant social media platforms to identify platform-specific patterns requiring tailored intervention. This gap is particularly consequential given emerging evidence that web-based information exposure influences cancer screening intentions and behaviors [21].

### Theoretical Framework

This study is guided by 3 complementary theoretical frameworks that inform the coding scheme and analytic approach. Table 1 provides explicit mapping from theoretical constructs to codebook items.

**Table 1.** Theory-to-codebook mapping.

Theory and construct	Codebook item	Operational definition
Diffusion of innovations		
Relative advantage	Benefits mentioned (D1.3)	Mortality reduction, early detection, cure potential mentioned
Compatibility	Values reference (D2.2)	References patient values, preferences, or life context
Complexity	Procedural clarity (D1.6)	Accurate description of LDCT^a^ procedure and what to expect
Observability	Survivor narrative (D1.7)	Personal success story or early detection outcome present
Attribution theory of stigma		
Controllability attribution	Explicit blame (D3.1)	Direct statements attributing illness to personal choice (“you did this”)
Responsibility judgment	Redemptive framing (D3.3)	Implies care/sympathy contingent on quitting (“if you quit, you deserve...")
Counter-attribution	Counter-stigma (D3.5)	Acknowledges addiction, structural factors, or challenges blame narratives
Health misinformation frameworks		
Accuracy	Clinical accuracy (D1.1‐1.5)	Eligibility criteria, procedure, outcomes verified against USPSTF^b^ guidelines
Completeness	Benefit-harm balance (D1.4)	Both benefits AND harms (false positives, radiation, anxiety) mentioned

a LDCT: low-dose computed tomography.

b USPSTF: United States Preventive Services Task Force.

1. Diffusion of innovations theory posits that adoption of new practices, including health behaviors such as cancer screening, is influenced by perceptions of the innovation’s characteristics [22]. Four constructs are particularly relevant: (1) relative advantage (perceived benefits compared with alternatives), (2) compatibility (alignment with values and existing practices), (3) complexity (perceived difficulty of understanding or undertaking the behavior), and (4) observability (visibility of outcomes). We operationalize these constructs to assess whether social media content communicates the advantages of LCS, addresses fit with patients’ lives and values, clarifies or complicates procedural understanding, and highlights screening success stories.
2. Attribution theory of stigma provides a framework for understanding how blame and responsibility attributions shape attitudes toward stigmatized conditions [23,24]. When disease is attributed to perceived controllable behaviors (such as smoking), individuals may be viewed as responsible for their illness and deserving of negative outcomes. This framework informs our coding of explicit and implicit blame, redemptive framing (suggesting that only those who quit “deserve” care), shame imagery, and counter-stigma messaging that challenges blame attributions.
3. Health misinformation frameworks describe how false or misleading health information spreads online and influences beliefs and behaviors [25,26]. Key constructs include accuracy (factual correctness), completeness (presence of important contextual information), source credibility, and emotional framing. We adapt these constructs to evaluate clinical accuracy of screening-related claims, balance in benefit-harm presentation, and the presence of misinformation about eligibility, procedures, or outcomes.

Together, these frameworks enable a comprehensive assessment of how social media content may facilitate or undermine informed screening decisions, perpetuate or challenge stigma, and align with or deviate from evidence-based guidance.

### Objectives

This study has 3 primary aims and one exploratory aim.

- Primary aim 1: Characterize the clinical accuracy, completeness, and decision-support quality of LCS-related content across platforms, including eligibility criteria, procedural information, and benefit-harm framing.
- Primary aim 2: Quantify the prevalence, forms, and severity of stigma, blame, and nihilism in lung cancer and LCS content, and identify platform-specific patterns.
- Primary aim 3: Test whether inaccurate or stigmatizing content is associated with disproportionate engagement compared with accurate, nonstigmatizing content, adjusting for platform, content type, and creator characteristics. Engagement metrics are treated as indicators of relative visibility and reach rather than direct measures of algorithmic amplification; observed associations may reflect algorithmic promotion, user preferences, content characteristics, or their interaction.
- Exploratory aim: Document representation patterns in visible content and identify digital opinion leaders (DOLs) who could serve as partners for evidence-based dissemination. DOLs will be categorized by creator type for inclusion in engagement regression models, enabling direct comparison of content characteristics between DOLs and other creators.

## Methods

### Study Design and Reporting

This is a cross-sectional content analysis of publicly accessible social media posts following established methodological standards for digital health research [27,28]. The protocol established methodological standards for social media research [29] and best practices for content analysis methodology [30]. The study protocol is registered on the Open Science Framework (https://osf.io/krq4h) to enhance transparency and reproducibility.

### Platforms

Seven platforms were selected based on user base size, demographic diversity, content modality, and relevance to health information seeking: (1) Facebook, (2) Instagram, (3) TikTok, (4) YouTube, (5) X/Twitter, (6) Reddit, and (7) Bluesky. LinkedIn was excluded due to its professional focus and minimal patient-generated content based on preliminary scoping; Threads was excluded due to insufficient lung cancer content volume at the time of protocol development. This selection captures a range of content formats (long-form video, short-form video, static images, and text-based discussion), algorithmic structures, and user communities. Bluesky is included as an emerging decentralized platform increasingly adopted by health communicators and researchers. We anticipate substantial variation in content volume, format, and community composition across platforms. Bluesky, as an emerging decentralized platform, is expected to yield fewer posts that are more likely news- or academic-oriented, whereas TikTok content is anticipated to be dominated by personal narratives and influencer-generated short-form video. These cross-platform differences are a central feature of the study design—the multiplatform approach is intended precisely to characterize how content and framing vary by platform architecture and community norms—but they may limit statistical power for platform-specific subgroup analyses on lower-volume platforms.

### Search Strategy

Search terms were developed through an iterative process involving four sources: (1) clinical guideline terminology from the USPSTF 2021 recommendation statement and associated clinical guidance; (2) prior social media research on lung cancer and cancer screening, from which we extracted search terms used in published studies [16-20]; (3) input from an expert panel comprising LCS researchers, a behavioral scientist specializing in stigma (LC-B), and a patient advocacy representative, who reviewed candidate terms for comprehensiveness and relevance; and (4) known stigma and nihilism signals identified from the lung cancer stigma literature [9-12], which informed the inclusion of Bundle B terms specifically designed to surface blame and fatalism narratives (eg, “I caused my lung cancer,” “smoking and lung cancer blame”). The final term list was refined during the preliminary scoping phase described above, with terms that yielded no relevant content or exclusively irrelevant content removed, and terms that surfaced unanticipated relevant content added. Terms are organized into 2 bundles corresponding to distinct content domains:

- Bundle A (LCS): “lung cancer screening,” “low-dose CT,” “LDCT,” “CT scan for lung cancer,” “lung cancer screening guidelines,” “Medicare lung cancer screening,” “should I get screened for lung cancer,” “lung cancer screening eligibility.”
- Bundle B (Lung Cancer Narratives): “lung cancer,” “lung cancer survivor,” “stage 4 lung cancer,” “non-small cell lung cancer,” “small cell lung cancer,” “quit smoking lung cancer,” “I caused my lung cancer,” “smoking and lung cancer blame.”

Bundle B includes terms specifically designed to surface stigma-laden content (eg, “I caused my lung cancer”) to ensure adequate representation of this construct for analysis.

### Search Execution

Search terms and the overall strategy were piloted across all 7 platforms during a preliminary scoping phase conducted in August-September 2025. This scoping assessed content volume, term relevance, and the degree to which search terms surfaced the target constructs (screening information, stigma/blame narratives, misinformation). Based on this scoping, we estimate a screening-to-inclusion ratio of approximately 2:1 to 3:1, meaning approximately 1500‐3000 posts will be screened to achieve the target sample of 700‐1000 eligible posts after applying exclusion criteria and deduplication. Should content volume fall below projections for specific platforms, we will use predefined contingency procedures: (1) extending the time window (eg, from 6 to 12 months for lower-volume platforms), (2) supplementing with additional related search terms identified during scoping, and (3) documenting any protocol modifications and their rationale in the final report. All contingency procedures and protocol deviations will be recorded and reported transparently. Search execution will use platform-native search interfaces accessed through standard web browsers in private/incognito mode without authentication to capture the public-view information environment.

Search execution will use platform-native search interfaces accessed through standard web browsers in private/incognito mode without authentication to capture the public-view information environment. This approach reflects real-world information-seeking behavior for users who are not logged in. Browsers will be configured with cleared cookies and cache to partially mitigate algorithmic personalization, though we acknowledge this approach captures the “default” algorithmic presentation rather than eliminating algorithmic influence entirely. For each platform, search queries will be entered verbatim; platform-specific syntax variations (eg, hashtag handling, quotation marks) will be documented in Multimedia Appendix 1 [4,27,28,30-33]. All searches will be conducted within a 72-hour window to minimize temporal variation. Search timestamps, query strings, result counts, and platform version information will be logged to support reproducibility and contextualization.

### Sampling Strategy

The sampling approach combines three complementary methods to capture diverse content types and maximize ecological validity [31]:

1. Relevance-based sampling: For each search term, the first 20 posts sorted by platform-default relevance ranking will be captured. This approach reflects the content most likely to be encountered by users conducting typical searches.
2. Engagement-based sampling: For each search term, the top 20 posts ranked by engagement metrics (likes, comments, shares, views) will be captured. This approach identifies high-visibility content that reaches the largest audiences.
3. Algorithmic recommendation sampling: For anchor terms (“lung cancer screening” and “lung cancer”), 5 posts surfaced through algorithmic recommendations (eg, “For You,” “Suggested,” “Up Next”) will be captured. This approach samples content actively promoted by platform algorithms.

Posts will be deduplicated within and across sampling methods. Based on preliminary scoping and precedent from similar multiplatform content analyses [32,33], we anticipate a final sample of approximately 700‐1000 unique posts.

This sample size was determined a priori through 3 considerations. First, for cross-platform comparisons (7 platforms), detecting medium effect sizes (Cohen w=0.30) in chi-square analyses with *α* values of 0.05% and 80% power requires approximately 108 posts per platform. Our target of 700‐1000 posts meets this threshold. Second, for regression modeling with approximately 12 predictors, a minimum of 10‐20 observations per predictor requires 120‐240 observations; our sample substantially exceeds this threshold. Third, content saturation in qualitative synthesis typically occurs by 100‐200 coded items in health communication research. We acknowledge that rare content types (eg, posts containing explicit eligibility misinformation) may have insufficient prevalence for subgroup analysis; such limitations will be reported transparently. The final sample size will be evaluated relative to both statistical requirements and practical content saturation; should saturation be reached earlier than anticipated or specific platforms yield lower-than-projected content volume, the final sample may fall at the lower end of the target range.

### Time Frame

Platform-specific time windows reflect content lifecycle differences. YouTube and Facebook content remains relevant longer; thus, posts from the preceding 12 months will be included. For faster-paced platforms (X/Twitter, Instagram, TikTok, Reddit, Bluesky), posts from the preceding 6 months will be included.

### Eligibility Criteria

The inclusion and exclusion criteria were as follows:

- Inclusion criteria: Publicly accessible posts in English containing substantive content about lung cancer or LCS (more than passing mention).
- Exclusion criteria: Private or members-only content; paid advertisements without educational content; non-English posts; duplicate reposts of identical content (shares and retweets are counted as engagement on the original post).

### Data Extraction

For each post, the following data will be extracted: platform; posting date; content type (video, image, text, infographic, link); creator type (patient/survivor, caregiver, clinician/health system, advocacy organization, commercial entity, influencer/general creator); sponsorship disclosures; engagement metrics (likes, comments, shares, views as available); and a paraphrased summary of content.

### Coding Framework

A structured codebook operationalizes the theoretical framework (Table 1) across 6 domains.

#### Domain 1: Clinical Accuracy and Completeness

For screening-focused posts, coders will assess whether LDCT is mentioned; whether eligibility criteria (age, smoking history) are stated and accurate; whether benefits (mortality reduction, early detection) are mentioned and appropriately framed; whether harms (false positives, incidental findings, radiation exposure, anxiety) are acknowledged; and whether overgeneralization occurs (eg, implying everyone should be screened). For general lung cancer posts, coders will assess mention of cancer type, stage, and prognosis accuracy, as well as the presence of medical misinformation (eg, unproven cures). A composite accuracy rating (mostly accurate, partially accurate, mostly inaccurate, cannot determine) will be assigned. Accuracy coding distinguishes three categories to prevent conflation of misinformation with opinion: (1) Factual claims about eligibility, procedures, or outcomes that can be verified against clinical guidelines (coded accurate, inaccurate, or incomplete); (2) Incomplete information that omits important context but contains no demonstrably false claims (coded “incomplete” rather than “inaccurate”); (3) Opinion or value statements that do not make verifiable factual claims (coded “not applicable for accuracy”). This taxonomy distinguishes legitimate patient preferences (eg, “I chose not to screen because the uncertainty stressed me”) from misinformation (eg, “Screening doesn’t reduce mortality”). Decision rules and boundary examples for each category are provided in Multimedia Appendix 2 [4,9,10,22-26,30,34].

#### Domain 2: Decision-Support Features

Informed by diffusion of innovations theory, coders will assess whether the post acknowledges choices or options; references values and preferences (“what matters to you”); provides appropriate next steps; and presents benefits and harms in a balanced manner. A decision-support strength score (0=none, 1=minimal/implicit, 2=explicit decision support) will be assigned.

#### Domain 3: Stigma, Blame, and Nihilism

Informed by attribution theory, coders will assess presence and intensity (none, mild, moderate, severe) of: explicit blame (“you did this to yourself”); implicit blame (emphasis on personal responsibility without acknowledging addiction or structural factors); redemptive framing (“if you quit, you deserve care”); nihilism (“lung cancer is always fatal,” “why bother screening”); counter-stigma messaging (“no one deserves lung cancer”); identity-first versus person-first language; and shame or disgust imagery. A composite stigma severity rating will be assigned.

#### Domain 4: Representation and Equity

Where discernible, coders will note apparent race/ethnicity, gender, and age of individuals depicted. Structural barrier mentions (insurance, transportation, rural access, language barriers, racism, mistrust) will be coded. The focus population (general, specific communities) will be documented. A conservative “not discernible” classification will be applied when demographic attributes cannot be reliably identified.

#### Domain 5: Tone and Emotional Framing

Coders will assess dominant emotional valence (hopeful, fearful, neutral) and presence of alarmism or sensationalism.

#### Domain 6: Engagement Metrics

Platform-specific engagement data will be standardized to enable cross-platform comparison. Posts will be categorized into engagement tertiles (high, medium, low) within each platform to account for differences in engagement scale across platforms.

### Composite Rating Construction and Reporting

Three composite ratings synthesize item-level assessments within their respective domains. The composite accuracy rating integrates item-level assessments across Domain 1 items (D1.1-D1.5). A post is rated “mostly accurate” when all verifiable claims align with USPSTF guidelines and no demonstrably false claims are present; “partially accurate” when the post contains a mix of accurate and inaccurate or incomplete claims; “mostly inaccurate” when the predominant claims are inconsistent with clinical evidence; and “cannot determine” when insufficient factual content is present for assessment. The composite stigma severity rating integrates Domain 3 items (D3.1-D3.5), ranging from “none” (no stigma indicators present) through “mild” (single implicit indicator), “moderate” (multiple indicators or one explicit indicator), to “severe” (explicit blame combined with nihilistic framing or shame imagery). The decision-support strength score (0‐2) is assigned based on the number and quality of decision-support features present across Domain 2 items: 0 indicates no decision-support features, 1 indicates minimal or implicit features, and 2 indicates explicit decision support. When item-level ratings within a composite do not converge clearly, coders will apply the decision rules detailed in Multimedia Appendix 2 [4,9,10,22-26,30,34], which include worked examples of boundary cases. Unresolved borderline cases will be flagged for adjudication by the principal investigator (LC-B), with reasoning documented in the Coding Decision Log. Additionally, sensitivity analyses described in the Statistical Analysis section include reclassification of borderline composite ratings to assess whether findings are robust to these judgment calls. Item-level distributions will be reported for all accuracy, stigma, and decision-support items alongside composite scores, enabling readers to identify which specific components drive composite patterns.

The complete codebook with operational definitions and decision rules is provided in Multimedia Appendix 2 [4,9,10,22-26,30,34].

### Coder Training and Reliability

A minimum of 2 coders will independently code each post. Coders will be research assistants with prior content analysis experience selected based on demonstrated attention to detail and familiarity with social media platforms.

Training will follow a structured 5-phase protocol:

1. Orientation (2 h): Overview of LCS clinical guidelines (USPSTF 2021), eligibility criteria, and stigma/nihilism concepts using published literature [9-12].
2. Codebook familiarization (2 h): Review of operational definitions with worked examples, boundary cases, and common pitfalls.
3. Calibration coding (4 h): Group coding of 20 posts stratified by platform with real-time discussion and codebook refinement based on encountered ambiguities.
4. Pilot reliability testing: Independent coding of 50 posts stratified by platform and content type. Gwet’s AC1 [34] will be calculated for each domain; coders achieving AC1≥0.70 on all domains proceed; others receive targeted retraining on low-reliability domains.
5. Drift monitoring: Every 100 coded posts, a random 10% subsample will be recoded by the alternate coder to detect coding drift. Drift exceeding 0.10 decline in AC1 from baseline will trigger a recalibration meeting with codebook clarification.

Discrepancies will be flagged in real-time using a shared coding database. Coders will attempt resolution through discussion within 24 hours. Unresolved discrepancies will be escalated to the principal investigator (LC-B) for final determination; all adjudication decisions and reasoning will be documented in a Coding Decision Log to ensure transparency and support future replication.

Interrater reliability will be calculated using Gwet’s AC1 [34] coefficient, which is preferred over Cohen kappa for content analysis due to its stability in the presence of prevalence imbalances and high agreement [34]. The threshold for acceptable reliability is AC1≥0.70 for accuracy and stigma domains. Percent agreement will be calculated for equity markers given the categorical and often conservative (“not discernible”) nature of these codes.

### Digital Opinion Leader Identification

As an exploratory aim, we will identify DOLs who influence public discourse around lung cancer and LCS. DOL candidates will be identified during content analysis based on creator characteristics and engagement patterns. DOLs will be categorized by creator type for inclusion in engagement regression models, enabling direct comparison of content characteristics (accuracy, stigma) between DOLs and other creators.

A structured scoring rubric (0‐9 points) will assess 5 criteria: (1) Credibility (0‐2 points): Verified status, professional credentials (clinician, researcher), or recognized organizational affiliation; (2) Reach (0‐2 points): Platform-specific follower/subscriber thresholds (eg, YouTube ≥5000; X/Twitter ≥10,000; TikTok ≥20,000); (3) Engagement rate (0‐2 points): Average engagement≥10%=2 points; 5%‐9%=1 point; (4) Relevance (0‐2 points): 5 or more lung cancer–focused posts in audit period=2 points; 2‐4 posts=1 point; and (5) Multiplatform presence (0‐1 point): Active on 2 or more platforms with relevant content.

DOLs will be categorized into tiers: Tier 1 (7‐9 points)=high priority for partnership; Tier 2 (4‐6 points)=moderate priority; Tier 3 (<4 points)=monitor only. The DOL roster will be validated with community partners and patient advisory groups. The complete scoring rubric is provided in Multimedia Appendix 3 [22,35-40]. We acknowledge that this scoring rubric is pragmatically derived for stakeholder mapping purposes and has not been psychometrically validated; the tiered roster represents a practical output for dissemination planning rather than a validated measurement instrument.

### Statistical Analysis

Descriptive analyses will summarize distributions of accuracy, stigma/nihilism, decision-support strength, equity markers, and engagement by platform, content domain (screening vs general lung cancer), and creator type.

Cross-platform comparisons will use chi-square or Fisher exact tests for categorical variables and Kruskal-Wallis tests for ordinal variables to identify significant differences in accuracy, stigma prevalence, and decision-support quality across platforms.

Engagement modeling will use negative binomial regression (for count outcomes) or ordinal logistic regression (for engagement tertiles) to test the hypothesis that content characteristics predict engagement. Predictors will include accuracy rating, stigma severity, creator type (including DOL status), content type, platform, and equity markers. This analysis will test whether inaccurate or stigmatizing content is associated with disproportionate engagement, as specified in Primary Aim 3. We note that significant associations between content characteristics and engagement should be interpreted as evidence of differential visibility rather than causal evidence of algorithmic bias, as multiple mechanisms may contribute to observed engagement patterns.

Subgroup analyses will compare screening-focused versus general lung cancer content; institutional versus individual creators; video versus static posts; and high- versus low-engagement content.

Sensitivity analyses will assess the robustness of findings to reclassification of borderline accuracy ratings, exclusion of posts with missing engagement data, normalization of engagement by time since posting, and comparison of content characteristics between relevance-based and algorithmic samples to assess potential algorithmic bias direction.

Qualitative synthesis will contextualize quantitative findings through thematic analysis of paraphrased content excerpts, identifying illustrative examples of key patterns.

### Ethical Considerations

This study was reviewed by the Hackensack Meridian Health Institutional Review Board and determined not to constitute regulated research involving human participants under 45 CFR 46.102(e)(1) as it involves secondary analysis of publicly available data with no direct interaction with individuals and no collection of identifiable private information.

While legally permissible, analysis of public health narratives raises ethical considerations beyond regulatory compliance. We adopt a minimization approach: (1) no direct quotations will appear in publications; content will be paraphrased and aggregated such that individual posters cannot be identified; (2) posts containing acute distress signals (eg, expressions of active suicidal ideation) will be excluded from analysis and not retained; and (3) the research team will not interact with or contact any content creators. These procedures exceed minimum regulatory requirements and align with emerging best practices for ethical social media research [27].

All procedures comply with the platform Terms of Service. No informed consent is required as the study uses only publicly posted content with no direct contact with social media users.

### Data Management

Data will be stored on institution-secured servers with role-based access restricted to approved research team members. The data management plan distinguishes three tiers: (1) raw data files containing post URLs and metadata will be retained for verification purposes but will not be shared publicly to comply with platform Terms of Service and protect user privacy; (2) analytic datasets will contain paraphrased content summaries, engagement metrics, and coded variables without direct identifiers linking to specific posts or users; and (3) aggregated summary datasets and the complete codebook will be prepared for public sharing. All data will be retained for 7 years following publication per institutional policy. Upon publication of results, the codebook, Coding Decision Log, aggregated summary statistics, and deidentified analytic datasets will be deposited in the Open Science Framework repository to support replication and secondary analysis.

## Results

Data collection began in October 2025, following finalization of the sampling framework and completion of coder training and is projected to be completed by July 2026. As of March 2026, data have been collected from 181 posts across 7 platforms. During the initial phase, posts were systematically identified and captured across all 7 platforms using the predefined search strategy. Dual coding commenced with weekly calibration meetings to ensure consistency in codebook application and maintain interrater reliability standards. Preliminary quality checks indicate strong coder alignment, with reliability metrics meeting prespecified thresholds.

Coding and adjudication will continue until the full dataset is complete. Formal quantitative analyses, including descriptive summaries, cross-platform comparisons, and regression modeling, will begin upon coding completion. The analytic phase is expected to conclude by mid-2026, with manuscript preparation and dissemination to follow.

## Discussion

### Principal Findings

This study addresses a critical gap in lung cancer prevention and early detection research. While substantial investment has been directed toward LCS trials, implementation science, and health system interventions, the digital information environment that shapes public understanding, fear, stigma, and nihilism has been largely unexamined. This protocol describes the first systematic, multiplatform, theory-informed analysis of lung cancer and LCS content on social media.

The study is expected to yield several novel contributions. First, by assessing accuracy across platforms and content types, findings will clarify the extent to which publicly visible LCS information aligns with guidelines, where inaccuracies cluster, and which creator types are most likely to disseminate misleading content. Second, the systematic application of a stigma and nihilism lens—operationalized through attribution theory—will provide the first empirical mapping of how blame, shame, and fatalism manifest and circulate in digital spaces. Third, the pairing of content characteristics with engagement metrics will illuminate whether stigmatizing or inaccurate content is associated with disproportionate engagement, which may reflect differential algorithmic visibility, user engagement preferences, or both, offering insights into the attention economy surrounding LCS. Fourth, documentation of representation patterns will quantify gaps in visibility for communities disproportionately affected by lung cancer but potentially underrepresented in online narratives. Importantly, interpretation of cross-platform differences will account for the inherent confounding between platform and content modality. Differences in stigma prevalence or accuracy between, for example, TikTok and Reddit may reflect the constraints and affordances of short-form video versus text-based discussion as much as community-level differences. The planned subgroup analysis comparing video versus static content will help disentangle modality effects from platform effects, though complete separation is not possible in an observational design.

### Comparison With Prior Work

Existing research on lung cancer content in social media has been limited in scope. Alban et al [16] analyzed YouTube videos related to lung cancer but did not assess stigma or decision-support features. Zhao et al [17] examined LCS information on social media but focused on a single platform. Our multiplatform approach, theory-informed codebook, and integration of stigma assessment with engagement dynamics extend this work substantially.

### Translational Applications

Findings will directly inform several ongoing initiatives. The characterization of dominant misconceptions, nihilistic narratives, and high-engagement misinformation will guide refinements to LungTalk, an evidence-based computer-tailored health communication and decision support tool for LCS. Real-world examples of stigmatizing content will be integrated into training modules for patient navigators and community health workers, enhancing their capacity to recognize and address digital influences on patient perceptions. The dual-coded, reliability-tested dataset will provide training examples that may inform future development of automated stigma detection tools. The labeled examples of explicit and implicit stigma in naturalistic social media contexts could serve as ground truth for supervised learning approaches. However, the feasibility of such applications depends on adequate representation of stigma-positive cases, class balance, and domain-specific model architecture considerations; this study is designed to characterize content patterns and is not intended to develop or validate automated detection systems. Finally, insights about platform-specific patterns will inform dissemination strategies for health systems and advocacy organizations seeking to reach eligible populations with accurate, stigma-informed messaging.

### Limitations

Several anticipated limitations warrant acknowledgment. First, the study characterizes the publicly visible information environment—the content most likely encountered by individuals conducting typical health information searches—rather than the totality of lung cancer discussions. This scope is intentional: publicly visible content shapes population-level exposure and is the appropriate target for public health communication interventions. Findings should not be generalized to private groups, direct messages, or content not surfaced by platform algorithms, which may differ substantially in tone and accuracy.

Second, the cross-sectional design captures a specific temporal window in a rapidly evolving digital environment. We address this limitation through three strategies: (1) documenting search timestamps, platform versions, and procedural details to enable contextualization of findings within the specific data collection period; (2) focusing on structural patterns (eg, stigma prevalence differences across platforms, relationship between accuracy and engagement) that are likely to be more stable than specific content; and (3) providing methodological infrastructure (codebook, sampling framework, analytic code) that enables future replication to track temporal trends. We position this study as establishing a baseline against which future shifts can be measured rather than claiming to capture a permanent state.

Third, representation coding is constrained by visual ambiguity; the conservative “not discernible” classification prevents inaccurate assumptions but may undercount certain demographic patterns. Fourth, the DOL scoring rubric is pragmatically derived for stakeholder mapping purposes and has not been psychometrically validated. Fifth, the inclusion of 7 platforms with substantially different content volumes may result in unequal sample sizes across platforms, particularly for Bluesky, which may yield fewer eligible posts. Should any platform produce fewer than the minimum posts needed for meaningful subgroup analysis, platform-specific findings will be interpreted descriptively rather than inferentially, and this will be noted transparently. Sixth, this study analyzes postlevel content and does not examine user comments or replies. Comment sections represent an important and often distinct dimension of public discourse, sentiment, and narrative framing—for example, stigmatizing or supportive responses to a patient’s personal narrative may substantially shape how that content is experienced by other viewers. Future research should examine comment-level content and sentiment to capture the full scope of public engagement with lung cancer and screening-related posts on social media. Despite these limitations, the systematic multiplatform design, dual-coding with reliability assessment, and theory-informed codebook collectively strengthen rigor.

### Conclusions

This protocol establishes a rigorous, reproducible framework for characterizing the digital information environment surrounding lung cancer and LCS. By integrating clinical accuracy assessment, stigma and nihilism coding, decision-support evaluation, and engagement analysis within a theory-informed design, the study will generate foundational evidence to guide communication interventions, navigator training, and equitable dissemination strategies. The methodology provides a replicable framework for monitoring health information ecosystems and is adaptable to other cancer types, screening modalities, and emerging platforms.

## Supplementary material

10.2196/89479 Multimedia Appendix 1 Platform-specific search parameters, syntax documentation, and data extraction templates.

10.2196/89479 Multimedia Appendix 2 Complete codebook with operational definitions, coding decision rules, and boundary case examples.

10.2196/89479 Multimedia Appendix 3 Digital opinion leader scoring rubric and identification procedures.

## Abbreviations

DOL: digital opinion leader
LCS: lung cancer screening
LDCT: low-dose computed tomography
USPSTF: United States Preventive Services Task Force
