# Supplementary material for: The Digital Information Environment of Lung Cancer and Lung Cancer Screening: Protocol for a Cross-Platform Social Media Content Analysis
Source: JMIR Res Protoc. 2026 Mar 30;15:e89479. doi: 10.2196/89479 (PMC13035028; doi:10.2196/89479)
Supplement: Multimedia Appendix 1 [file resprot-v15-e89479-s001.docx]

**Multimedia Appendix 1**

Platform-Specific Search Parameters and Data Extraction Templates

# **1. Search Term Bundles**

Search terms were developed based on clinical guidelines, prior literature, and stigma signals identified in preliminary scoping [1,2].

## ***1.1 Bundle A: Lung Cancer Screening***

**Primary terms:** lung cancer screening, low-dose CT, LDCT, CT scan for lung cancer, lung cancer screening guidelines, Medicare lung cancer screening, should I get screened for lung cancer, lung cancer screening eligibility

**Hashtag variants:** #lungcancerscreening, #LDCT, #lungscreening, #getchecked, #earlydetection, #lungcancerawareness, #screeningsaveslives, #knowyourrisks

## ***1.2 Bundle B: Lung Cancer Narratives***

**Primary terms:** lung cancer, lung cancer survivor, stage 4 lung cancer, non-small cell lung cancer, small cell lung cancer, quit smoking lung cancer, I caused my lung cancer, smoking and lung cancer blame

**Hashtag variants:** #lungcancer, #lungcancersurvivor, #lungcancerawareness, #cancerwarrior, #nevergiveup, #cancersurvivor, #fightcancer, #nobodydeserveslungcancer

# **2. Platform-Specific Search Parameters**

## ***2.1 YouTube***

| **Parameter** | **Specification** |
| --- | --- |
| Time window | 12 months preceding data collection |
| Filters | Upload date: This year; Type: Video; Features: Subtitles/CC (optional) |
| Sort options | Relevance (default), View count (for engagement sampling) |
| Minimum threshold | ≥1,000 views |
| Data captured | Video title, channel name, view count, likes, comments, upload date, video duration, description (first 500 characters) |

## ***2.2 Facebook***

| **Parameter** | **Specification** |
| --- | --- |
| Time window | 12 months preceding data collection |
| Filters | Public posts only; Posts from Pages, Groups (public), and Public profiles |
| Sort options | Top Posts (default), Recent Posts |
| Exclusions | Private groups, friends-only posts, ads without educational content |
| Data captured | Post text, page/profile name, reactions, comments, shares, post date, media type |

## ***2.3 Instagram***

| **Parameter** | **Specification** |
| --- | --- |
| Time window | 6 months preceding data collection |
| Search method | Hashtag search and keyword search |
| Tabs | Top (for engagement sampling), Recent (for relevance sampling) |
| Content types | Feed posts, Reels, Carousels |
| Data captured | Caption text, account name, likes, comments, post date, media type, follower count |

## ***2.4 TikTok***

| **Parameter** | **Specification** |
| --- | --- |
| Time window | 6 months preceding data collection |
| Search method | Keyword search and hashtag search |
| Filters | Top (most liked), Most Liked, Newest |
| Content types | Videos only |
| Data captured | Video caption, creator name, likes, comments, shares, views, post date, video duration, follower count |

## ***2.5 X/Twitter***

| **Parameter** | **Specification** |
| --- | --- |
| Time window | 6 months preceding data collection |
| Search syntax | Advanced Search: "lung cancer" OR "lung cancer screening" lang:en -is:retweet |
| Filters | Top (for engagement), Latest (for relevance) |
| Exclusions | Retweets (counted as engagement only), promotional accounts |
| Data captured | Tweet text, username, likes, retweets, replies, quote tweets, post date, follower count, verified status |

## ***2.6 Reddit***

| **Parameter** | **Specification** |
| --- | --- |
| Time window | 6 months preceding data collection |
| Target subreddits | r/lungcancer, r/cancer, r/stopsmoking, r/askdocs, r/health, r/medicine |
| Search method | Subreddit-specific search and site-wide search |
| Sort options | Top (past 6 months), Hot, Relevance |
| Data captured | Post title, post body, subreddit, upvotes, comments, post date, author karma |

## ***2.7 Bluesky***

| **Parameter** | **Specification** |
| --- | --- |
| Time window | 6 months preceding data collection |
| Search method | Native search function, hashtag search |
| Sort options | Top, Latest |
| Data captured | Post text, handle, likes, reposts, replies, post date, follower count |

# **3. Sampling Summary by Platform**

Based on methodological precedents for social media content analysis [3,4].

| **Platform** | **Relevance (20/term)** | **Engagement (20/term)** | **Algorithmic (5/anchor)** | **Est. Total** |
| --- | --- | --- | --- | --- |
| YouTube | 320 | 320 | 10 | ~400* |
| Facebook | 320 | 320 | 10 | ~400* |
| Instagram | 320 | 320 | 10 | ~400* |
| TikTok | 320 | 320 | 10 | ~400* |
| X/Twitter | 320 | 320 | 10 | ~400* |
| Reddit | 320 | 320 | 10 | ~400* |
| Bluesky | 320 | 320 | 10 | ~400* |

**Estimated after deduplication within platform. Cross-platform deduplication will further reduce total to estimated 700-1,000 unique posts.*

# **4. Data Extraction Template Fields**

The data extraction template (Excel workbook) contains the following field categories. Complete operational definitions are provided in Multimedia Appendix 1.

## ***4.1 Post Identification***

Post_ID, Platform, Post_URL, Collection_Date, Post_Date, Sampling_Method, Search_Term

## ***4.2 Content Characteristics***

Content_Format, Content_Domain, Creator_Type, Sponsorship, Video_Duration_Sec

## ***4.3 Engagement Metrics***

Likes, Comments, Shares_Reposts, Views, Upvotes, Days_Since_Post

## ***4.4 Clinical Accuracy Fields***

LDCT_Mentioned, Eligibility_Accuracy, Benefits_Framing, Harms_Framing, Overgeneralization, Cancer_Type, Misinformation_Present, Composite_Accuracy

## ***4.5 Decision Support Fields***

Relative_Advantage, Compatibility, Complexity, Choices_Acknowledged, Values_Referenced, Next_Steps, Decision_Support_Score

## ***4.6 Stigma Fields***

Explicit_Blame, Implicit_Blame, Redemptive_Framing, Nihilism, Counter_Stigma, Identity_Language, Shame_Imagery, Stigma_Severity

## ***4.7 Representation Fields***

Apparent_Race, Apparent_Gender, Apparent_Age, Structural_Barriers, Focus_Population

## ***4.8 Qualitative/Administrative Fields***

Content_Summary, Qualitative_Notes, Coder_1_ID, Coder_2_ID, Discrepancy_Flag, Resolution_Notes, DOL_Candidate

# **5. Data Management Procedures**

**File naming:** LCS_[Platform]_[Date]_[CoderInitials].xlsx

**Version control:** All changes logged in Changelog tab with date, editor, and description

**Quality assurance:** Weekly completeness checks, range validation, duplicate identification

**Privacy protection:** Post URLs stored separately from analytic dataset; no usernames in final dataset

**Backup:** Daily automatic backup to secure cloud storage

**Template Version:** 1.0

**Date:** October 2025

# **References**

1. Reuter K, Wilson ML, Gueye J, et al. Methods for systematically searching for and identifying health information on social media: scoping review. J Med Internet Res. 2021;23(12):e30459. [doi: 10.2196/30459] [PMID: 34941545]

2. Sinnenberg L, Buttenheim AM, Padrez K, Mancheno C, Ungar L, Merchant RM. Twitter as a tool for health research: a systematic review. Am J Public Health. 2017;107(1):e1-e8. [doi: 10.2105/AJPH.2016.303512] [PMID: 27854532]

3. Chen L, Wang X, Peng TQ. Nature and diffusion of gynecologic cancer-related misinformation on social media: analysis of tweets. J Med Internet Res. 2018;20(10):e11515. [doi: 10.2196/11515] [PMID: 30373727]

4. Basch CH, Kecojevic A, Wagner VH. Coverage of the COVID-19 pandemic in the online versions of highly circulated US daily newspapers. J Community Health. 2020;45(6):1089-1097. [doi: 10.1007/s10900-020-00913-4] [PMID: 32902813]

5. US Preventive Services Task Force. Screening for lung cancer: US Preventive Services Task Force recommendation statement. JAMA. 2021;325(10):962-970. [doi: 10.1001/jama.2021.1117] [PMID: 33687470]

6. Eysenbach G, Till JE. Ethical issues in qualitative research on internet communities. BMJ. 2001;323(7321):1103-1105. [doi: 10.1136/bmj.323.7321.1103] [PMID: 11701577]

7. Krippendorff K. Content Analysis: An Introduction to Its Methodology. 4th ed. Sage Publications; 2018. [ISBN: 978-1506395661]
