# Supplementary material for: The Digital Information Environment of Lung Cancer and Lung Cancer Screening: Protocol for a Cross-Platform Social Media Content Analysis
Source: JMIR Res Protoc. 2026 Mar 30;15:e89479. doi: 10.2196/89479 (PMC13035028; doi:10.2196/89479)
Supplement: Multimedia Appendix 2 [file resprot-v15-e89479-s002.docx]

Multimedia Appendix 2

Complete Codebook with Operational Definitions and Coding Decision Rules

# 1. Overview and General Instructions

This codebook provides operational definitions for coding social media content related to lung cancer and lung cancer screening (LCS). All codes are informed by Diffusion of Innovations theory [1], attribution theory of stigma [2,3], and health misinformation frameworks [4,5].

**General coding rules:**

- Code based on manifest content (what is explicitly stated or shown)
- When uncertain between two codes, choose the more conservative option
- Use "Cannot determine" when insufficient information exists
- All posts receive dual coding; discrepancies resolved through consensus

# 2. Domain 1: Content Characteristics

## 2.1 Platform

Record the platform where the post was captured: Facebook, Instagram, TikTok, YouTube, X/Twitter, Reddit, or Bluesky.

## 2.2 Content Format

- Video (short-form <60 seconds, long-form ≥60 seconds)
- Image/Photo (single or carousel)
- Text-only
- Infographic
- Link/article share
- Mixed (multiple formats)

## 2.3 Creator Type

- **Patient/survivor:** Individual sharing personal lung cancer experience
- **Caregiver/family member:** Individual sharing experience caring for someone with lung cancer
- **Clinician/health system:** Licensed healthcare provider or healthcare organization
- **Advocacy organization:** Nonprofit focused on lung cancer or cancer generally
- **Commercial entity:** For-profit company (pharma, screening facility, etc.)
- **Influencer/general creator:** Content creator without specific lung cancer credentials
- **News/media outlet:** Journalistic organization
- **Cannot determine:** Insufficient information to classify

## 2.4 Content Domain

- **LCS-focused:** Primary focus on lung cancer screening
- **General lung cancer:** Focus on diagnosis, treatment, survivorship, or general awareness
- **Mixed:** Addresses both screening and general lung cancer content

## 2.5 Sponsorship

- **Disclosed sponsorship:** Clear indication of paid partnership or sponsorship
- **Undisclosed/possible:** Appears promotional without disclosure
- **None apparent:** No indication of sponsorship

# 3. Domain 2: Clinical Accuracy and Completeness

This domain assesses the factual accuracy of medical and health information in posts. Accuracy coding is informed by health misinformation frameworks [4,5] and requires distinguishing among factual claims, incomplete information, and opinion/value statements.

## 3.1 Claim Type Classification

Before assessing accuracy, coders must first classify the type of claim being made. This taxonomy prevents conflation of misinformation with legitimate patient preferences or incomplete but non-misleading content.

### 3.1.1 Classification Categories

**Category 1: Factual Claims**

**Definition:** Statements about eligibility criteria, medical procedures, outcomes, or other verifiable facts that can be assessed against clinical guidelines or scientific evidence.

**Coding:** Code as Accurate, Inaccurate, or Incomplete (see Section 3.1.2)

*Examples of factual claims:*

- "Lung cancer screening is recommended for adults 50-80 with a smoking history" → *Factual claim (verify against USPSTF guidelines)*
- "Screening reduces lung cancer deaths by 20%" → *Factual claim (accurate per NLST)*
- "The scan uses radiation" → *Factual claim (accurate)*
- "Screening prevents lung cancer" → *Factual claim (inaccurate—screening detects, does not prevent)*
- "Everyone should get screened for lung cancer" → *Factual claim (inaccurate—only high-risk individuals)*

**Category 2: Incomplete Information**

**Definition:** Statements that are factually correct as stated but omit important contextual information that could lead to misunderstanding. The statement contains no demonstrably false claims.

**Coding:** Code as Incomplete. Do NOT code as Inaccurate.

**Key distinction:** Incomplete information is missing context; inaccurate information contains demonstrably false claims.

*Examples of incomplete information:*

- "Lung cancer screening can save lives" (true, but doesn't mention eligibility criteria) → *Incomplete*
- "The screening test is a CT scan" (true, but doesn't specify low-dose) → *Incomplete*
- "Screening has benefits" (true, but doesn't mention harms) → *Incomplete*
- "Talk to your doctor if you're worried about lung cancer" (true, but no eligibility guidance) → *Incomplete*

**Category 3: Opinion or Value Statements**

**Definition:** Statements expressing personal preferences, values, emotions, or subjective experiences that do not make verifiable factual claims about medical information.

**Coding:** Code as Not Applicable for Accuracy. These statements cannot be assessed for factual correctness.

**Key distinction:** Opinions express how someone feels or what they prefer; they are not claims about objective medical facts.

*Examples of opinion/value statements:*

- "I chose not to get screened because the uncertainty would stress me out" → *Opinion (personal preference)*
- "The waiting was the hardest part" → *Opinion (subjective experience)*
- "I'd rather not know" → *Opinion (personal value)*
- "Getting screened gave me peace of mind" → *Opinion (subjective experience)*
- "I think everyone should consider their family history" → *Opinion (personal recommendation)*

### 3.1.2 Decision Rules for Claim Classification

| **If the statement...** | **Then classify as...** | **And code accuracy as...** |
| --- | --- | --- |
| Makes a claim that can be verified against guidelines or evidence | Factual Claim | Accurate, Inaccurate, or Incomplete |
| Is factually correct but missing important context | Incomplete Information | Incomplete |
| Expresses feelings, preferences, or subjective experiences | Opinion/Value Statement | Not Applicable |
| Contains both factual claims AND opinions | Mixed | Assess factual claims only; note opinions separately |

### 3.1.3 Boundary Examples: Opinion vs. Misinformation

The most critical distinction is between legitimate patient preferences (opinions) and misinformation (false factual claims). The following examples illustrate this boundary:

| **Statement** | **Classification** | **Rationale** |
| --- | --- | --- |
| "I chose not to get screened because the anxiety wasn't worth it for me." | Opinion | Personal value judgment about trade-offs; no factual claim |
| "Screening doesn't reduce lung cancer deaths." | Misinformation | Verifiable factual claim that is demonstrably false |
| "I don't trust the medical system." | Opinion | Personal feeling; no factual claim about screening |
| "Doctors push screening to make money, not because it helps." | Misinformation | Implies false factual claim that screening doesn't help |
| "The scan was quick and painless." | Opinion | Subjective personal experience |
| "The scan takes about 10-15 minutes." | Factual Claim | Verifiable; code as Accurate |
| "For me, the peace of mind was worth the radiation exposure." | Opinion | Personal value trade-off |
| "The radiation from screening causes cancer." | Misinformation | Exaggerated factual claim; LDCT radiation risk is minimal |
| "Screening can find lung cancer early." | Incomplete | True but omits eligibility criteria |

### 3.1.4 Complex Cases: Mixed Content

Many posts contain both factual claims and opinions. In such cases, separately assess each component:

**Example 1:** "Screening reduced my risk of dying from lung cancer, and I'm so grateful I did it."

- "Screening reduced my risk of dying" → Factual claim (partially accurate—screening doesn't reduce individual risk, it enables early detection)
- "I'm so grateful" → Opinion (not coded for accuracy)

**Coding:** Code composite accuracy as Partially Accurate based on the factual claim component

**Example 2:** "The screening process was uncomfortable for me, but it only takes a few seconds of holding your breath."

- "Was uncomfortable for me" → Opinion (subjective experience)
- "Takes a few seconds of holding your breath" → Factual claim (accurate)

**Coding:** Code composite accuracy as Mostly Accurate based on the factual claim component

**Example 3:** "I didn't get screened because I heard it doesn't really work anyway, and honestly I'd rather not know."

- "It doesn't really work" → Factual claim (inaccurate misinformation)
- "I'd rather not know" → Opinion (personal preference)

**Coding:** Code composite accuracy as Mostly Inaccurate due to the misinformation component, even though an opinion is also present

***3.2 Screening-Specific Accuracy Items (LCS-focused posts only)***

**LDCT mentioned:** Yes / No / Not applicable

Does the post mention low-dose CT as the screening modality? Code "No" if only "CT scan" or "screening" is mentioned without specifying low-dose.

**Eligibility accuracy:** Accurate / Partially accurate / Inaccurate / Incomplete / Not mentioned

Per current USPSTF guidelines [6]: age 50-80, 20+ pack-year history, current smoker or quit within 15 years.

- **Accurate:** Correctly states all mentioned criteria
- **Partially accurate:** Some criteria correct, some incorrect (e.g., correct age but wrong pack-year threshold)
- **Inaccurate:** Criteria stated are wrong (e.g., "screening is for everyone")
- **Incomplete:** Mentions screening without specifying who qualifies
- **Not mentioned:** Eligibility criteria not addressed

**Benefits framing:** Balanced / Overstated / Understated / Incomplete / Not mentioned

- **Balanced:** Mentions mortality reduction with appropriate magnitude (~20%)
- **Overstated:** Implies screening prevents all lung cancer deaths or guarantees survival
- **Understated:** Minimizes proven benefits
- **Incomplete:** Mentions benefits vaguely without specifics ("can help")

**Harms framing:** Balanced / Overstated / Understated / Incomplete / Not mentioned

- **Balanced:** Acknowledges false positives, follow-up procedures, or anxiety without excessive alarm
- **Overstated:** Exaggerates risks ("radiation causes cancer")
- **Understated:** Claims screening is "completely safe" with no downsides

**Overgeneralization:** Present / Absent

Present if post implies everyone should be screened regardless of eligibility (e.g., "everyone over 40 needs this scan").

## 3.3 General Lung Cancer Accuracy Items

**Cancer type specified:** NSCLC / SCLC / Unspecified / Other

**Stage mentioned:** Yes (specify stage) / No

**Prognosis accuracy:** Accurate / Inaccurate / Not mentioned

If survival statistics or prognosis information is mentioned, assess against current data. Note that prognosis varies substantially by stage.

**Medical misinformation present:** Yes / No

Yes if post contains demonstrably false medical claims such as unproven cures, incorrect survival statistics, or false causation claims (e.g., "lung cancer is caused by cell phones").

## 3.4 Composite Accuracy Rating

Based on all accuracy items assessed, assign one overall rating:

- **Mostly accurate:** All verifiable factual claims are correct; appropriate framing of benefits/harms; no misinformation
- **Partially accurate:** Mix of accurate and inaccurate elements; OR accurate information with misleading framing; OR factual claims with significant incompleteness that could mislead
- **Mostly inaccurate:** Majority of verifiable claims are false or misleading; OR contains serious misinformation that could cause harm
- **Cannot determine:** Insufficient factual claims to assess; OR post contains only opinion/value statements (code claim type as Not Applicable)

# 4. Domain 3: Decision-Support Features

Informed by Diffusion of Innovations theory [1], this domain assesses whether content supports informed decision-making about lung cancer screening.

## 4.1 Relative Advantage (0-2)

Does the post communicate benefits of LCS compared to no screening?

- **0 = Not mentioned**
- **1 = Minimal** (vague mention of "catching cancer early")
- **2 = Explicit** (specific benefits like mortality reduction, earlier stage at diagnosis)

## 4.2 Compatibility (0-2)

Does the post address how screening fits with patients' values, beliefs, or lifestyle?

- **0 = Not mentioned**
- **1 = Minimal** (acknowledges screening is a personal choice)
- **2 = Explicit** (discusses values like peace of mind, family responsibility, personal autonomy)

## 4.3 Complexity (0-2)

Does the post clarify or simplify screening procedures?

- **0 = Not mentioned or increases confusion**
- **1 = Minimal** (brief mention of what to expect)
- **2 = Explicit** (clear explanation of process, duration, what happens during/after)

## 4.4 Choices Acknowledged (0-2)

Does the post acknowledge that screening is a choice?

- **0 = Screening presented as mandatory or only option**
- **1 = Implicit acknowledgment of choice**
- **2 = Explicit discussion of shared decision-making**

## 4.5 Values Referenced (0-2)

Does the post reference personal values in decision-making?

- **0 = Not mentioned**
- **1 = Minimal** ("talk to your doctor about what's right for you")
- **2 = Explicit** (discusses weighing pros/cons based on personal priorities)

## 4.6 Next Steps Provided (0-2)

Does the post provide actionable next steps?

- **0 = No action items**
- **1 = Vague** ("talk to your doctor")
- **2 = Specific** (provides eligibility checker link, screening locator, or specific conversation starters)

## 4.7 Decision-Support Score

Sum of items 4.1-4.6 (range 0-12). Categorize as: **None** (0), **Minimal** (1-4), **Moderate** (5-8), **Strong** (9-12).

# 5. Domain 4: Stigma, Blame, and Nihilism

Informed by attribution theory of stigma [2,3], this domain assesses how content perpetuates or challenges lung cancer stigma.

## 5.1 Explicit Blame

Direct statements attributing lung cancer to personal choices or holding individuals responsible.

- **None:** No blame language
- **Mild:** Subtle suggestions ("lifestyle choices have consequences")
- **Moderate:** Clear attribution ("smoking caused your cancer")
- **Severe:** Harsh judgment ("you did this to yourself," "you deserve this")

*Example (Severe):* "I have no sympathy for smokers who get lung cancer. They knew the risks."

## 5.2 Implicit Blame

Emphasis on personal responsibility without acknowledging addiction, structural factors, or never-smoker cases.

- **None:** No implicit blame
- **Mild:** Overemphasis on personal choice without context
- **Moderate:** Frames lung cancer as entirely preventable
- **Severe:** Systematic attribution to individual behavior while ignoring other factors

*Example (Moderate):* "Lung cancer is 100% preventable if you just don't smoke."

## 5.3 Redemptive Framing

Implies that only those who quit smoking or reformed deserve care/sympathy.

- **Present:** Conditional worth based on behavior change
- **Absent:** No redemptive framing

*Example (Present):* "She quit 10 years ago, so she doesn't deserve this."

## 5.4 Nihilism

Fatalistic beliefs that lung cancer is uniformly fatal or that screening/treatment is futile.

- **None:** No nihilistic content
- **Mild:** Pessimistic tone without explicit fatalism
- **Moderate:** Statements implying poor outcomes are inevitable
- **Severe:** Explicit statements that all lung cancer patients die or that treatment is pointless

*Example (Severe):* "Lung cancer is basically a death sentence. Why bother with screening?"

## 5.5 Counter-Stigma Messaging

Content that explicitly challenges stigma, blame, or nihilism.

- **Present:** Actively challenges stigma ("No one deserves lung cancer," "Lung cancer affects never-smokers too")
- **Absent:** No counter-stigma content

*Example (Present):* "Stop asking lung cancer patients if they smoked. It doesn't matter. They deserve compassion."

## 5.6 Identity Language

- **Person-first:** "Person with lung cancer"
- **Identity-first:** "Lung cancer patient" (neutral unless used disparagingly)
- **Stigmatizing labels:** "Smoker," "victim" (when used pejoratively)
- **Not applicable:** No individuals referenced

## 5.7 Shame Imagery

Visual or verbal content intended to evoke shame or disgust.

- **Present:** Graphic imagery, shaming language, disgust cues
- **Absent:** No shame imagery

## 5.8 Stigma Severity Composite

Overall stigma assessment based on items 5.1-5.7:

- **None:** No stigmatizing elements; may include counter-stigma
- **Low:** Minor stigmatizing elements without severe blame or nihilism
- **Moderate:** Clear stigmatizing content in multiple domains
- **High:** Severe blame, nihilism, or multiple stigmatizing elements

# 6. Domain 5: Representation and Equity

## 6.1 Apparent Race/Ethnicity

Code based on visual appearance when discernible. Use conservative coding.

**Options:** White, Black/African American, Hispanic/Latino, Asian, Other/Multiple, Not discernible

## 6.2 Apparent Gender

**Options:** Male, Female, Non-binary/Other, Multiple, Not discernible

## 6.3 Apparent Age Group

**Options:** Young adult (18-39), Middle-aged (40-64), Older adult (65+), Multiple ages, Not discernible

## 6.4 Structural Barriers Mentioned

Code all that apply:

- Insurance/cost barriers
- Transportation barriers
- Rural/geographic access
- Language barriers
- Medical mistrust/racism
- Occupational exposure
- Environmental exposure (radon, air quality)
- None mentioned

## 6.5 Focus Population

- General audience
- Veterans
- Black/African American communities
- Rural populations
- Never-smokers
- Women
- Other specific population (specify)

# 7. Domain 6: Tone and Emotional Framing

## 7.1 Emotional Valence

- **Hopeful/Positive:** Emphasizes survival, treatment advances, empowerment
- **Fearful/Negative:** Emphasizes death, suffering, fear
- **Neutral/Informational:** Factual without strong emotional framing
- **Sad/Grief:** Memorial content, mourning
- **Angry/Advocacy:** Calling out injustice, demanding change
- **Mixed:** Multiple emotional tones

## 7.2 Alarmism/Sensationalism

- **Present:** Exaggerated claims, clickbait language, excessive fear appeals
- **Absent:** Appropriate tone for content

# 8. Domain 7: Engagement Metrics

Record platform-specific engagement metrics as available:

- Likes/Reactions (all platforms)
- Comments (all platforms)
- Shares/Reposts (Facebook, X, TikTok)
- Views (YouTube, TikTok, Instagram Reels)
- Upvotes/Downvotes (Reddit)
- Days since posting (calculate)

Engagement tertiles will be calculated within platform during analysis.

# 9. Composite Ratings Summary

At completion of coding, assign the following composite ratings:

1. **Claim Type Classification** (Factual Claim / Incomplete Information / Opinion or Value Statement / Mixed / Cannot Determine)
2. **Composite Accuracy Rating** (Mostly accurate / Partially accurate / Mostly inaccurate / Cannot determine)
3. **Decision-Support Score** (0-12; categorized as None / Minimal / Moderate / Strong)
4. **Stigma Severity** (None / Low / Moderate / High)
5. **DOL Candidate Flag** (Yes / No)

# 10. Coding Decision Rules and Edge Cases

## 10.1 General Decision Rules

1. When in doubt, choose the more conservative code.
2. Code manifest content only; do not infer intent.
3. If multiple people appear, code the primary subject.
4. Treat emojis as content when relevant to coding.
5. Code captions and video content together as a unit.
6. For mixed content (factual claims + opinions), assess factual claims for accuracy and note opinions separately.

## 10.2 Common Edge Cases

**Edge case:** Post mentions "getting checked" without specifying LDCT.

**Decision:** Code LDCT mentioned as "No" unless LDCT/low-dose CT is explicitly stated.

**Edge case:** Commercial post promoting screening services.

**Decision:** Include if educational content present; code creator type as Commercial; note sponsorship.

**Edge case:** Post mentions smoking history without blame language.

**Decision:** Factual mention of smoking is not automatically implicit blame. Code as No Implicit Blame unless responsibility/judgment is implied.

**Edge case:** Survivor story mentions smoking factually.

**Decision:** Code implicit blame as Absent. Factual mention of personal history is not blame.

**Edge case:** Post uses outdated eligibility criteria.

**Decision:** Code as Partially accurate with note. Criteria were correct previously but are now outdated.

**Edge case:** Memorial post for someone who died of lung cancer.

**Decision:** Include if contains substantive content about lung cancer. Code tone as Sad/Grief. For accuracy, code as Cannot Determine if no factual claims present.

**Edge case:** Post mentions radon or environmental causes.

**Decision:** Include. May be coded as counter-stigma if challenging smoking-only narrative.

**Edge case:** Race/ethnicity unclear from visual content.

**Decision:** Code as "Not discernible." Do not guess.

**Edge case:** Post in comments thread, not original post.

**Decision:** Generally exclude. Code only original posts unless comment is substantive and widely engaged.

**Edge case:** Repost/share of another creator's content.

**Decision:** Code the original content. Record engagement on repost as engagement data.

**Edge case:** Post expresses personal preference that could discourage screening.

**Decision:** Distinguish opinion from misinformation. "I'd rather not know" = Opinion (Not Applicable for accuracy). "Screening is pointless" = Misinformation (Inaccurate). Personal preferences are not coded as misinformation even if they result in not screening.

**Edge case:** Post contains both accurate information AND opinion that discourages screening.

**Decision:** Assess factual claims separately from opinions. A post can be Mostly Accurate on facts while also expressing a personal preference against screening. Note the presence of both in comments.

## 10.3 Coding Decision Log

All difficult coding decisions should be recorded in the Coding Decision Log with: Post ID, Variable(s) in question, Decision made, Rationale, Coder initials, and Date. Logged decisions will be reviewed weekly during calibration meetings.

**Codebook Version:** 1.0

**Date:** October 2025

# References

1. Rogers EM. Diffusion of Innovations. 5th ed. Free Press; 2003. [ISBN: 978-0743222099]

2. Weiner B, Perry RP, Magnusson J. An attributional analysis of reactions to stigmas. J Pers Soc Psychol. 1988;55(5):738-748. [doi: 10.1037/0022-3514.55.5.738] [PMID: 3210143]

3. Link BG, Phelan JC. Conceptualizing stigma. Annu Rev Sociol. 2001;27:363-385. [doi: 10.1146/annurev.soc.27.1.363]

4. Wang Y, McKee M, Torbica A, Stuckler D. Systematic literature review on the spread of health-related misinformation on social media. Soc Sci Med. 2019;240:112552. [doi: 10.1016/j.socscimed.2019.112552] [PMID: 31561111]

5. Suarez-Lledo V, Alvarez-Galvez J. Prevalence of health misinformation on social media: systematic review. J Med Internet Res. 2021;23(1):e17187. [doi: 10.2196/17187] [PMID: 33470931]

6. US Preventive Services Task Force. Screening for lung cancer: US Preventive Services Task Force recommendation statement. JAMA. 2021;325(10):962-970. [doi: 10.1001/jama.2021.1117] [PMID: 33687470]

7. Hamann HA, Ostroff JS, Marks EG, Gerber DE, Schiller JH, Lee SJC. Stigma among patients with lung cancer: a patient-reported measurement model. Psychooncology. 2014;23(1):81-92. [doi: 10.1002/pon.3371] [PMID: 24038640]

8. Chambers SK, Dunn J, Occhipinti S, et al. A systematic review of the impact of stigma and nihilism on lung cancer outcomes. BMC Cancer. 2012;12:184. [doi: 10.1186/1471-2407-12-184] [PMID: 22587342]

9. Krippendorff K. Content Analysis: An Introduction to Its Methodology. 4th ed. Sage Publications; 2018. [ISBN: 978-1506395661]

10. Gwet KL. Computing inter-rater reliability and its variance in the presence of high agreement. Br J Math Stat Psychol. 2008;61(Pt 1):29-48. [doi: 10.1348/000711006X126600] [PMID: 18482474]
