# Supplementary material for: The Digital Information Environment of Lung Cancer and Lung Cancer Screening: Protocol for a Cross-Platform Social Media Content Analysis
Source: JMIR Res Protoc. 2026 Mar 30;15:e89479. doi: 10.2196/89479 (PMC13035028; doi:10.2196/89479)
Supplement: Multimedia Appendix 3 [file resprot-v15-e89479-s003.docx]

**Multimedia Appendix 3**

Digital Opinion Leader Scoring Rubric and Identification Procedures

# **1. Purpose**

This rubric provides standardized criteria for identifying and scoring Digital Opinion Leaders (DOLs) who influence public discourse around lung cancer and lung cancer screening (LCS) on social media. DOLs are individuals or organizations whose content reaches substantial audiences and shapes public understanding, attitudes, and behaviors related to lung cancer [1,2].

**Intended uses:**

• Identify potential partners for evidence-based health communication campaigns

• Characterize the landscape of influential voices in lung cancer discourse

• Support targeted dissemination of stigma-reduction messaging

• Inform community engagement strategies

# **2. DOL Identification Procedures**

DOL candidates are identified through three complementary approaches:

**2.1 Content Analysis Discovery:** During coding of sampled posts, coders flag creators who meet preliminary criteria (high engagement, multiple relevant posts, apparent credibility).

**2.2 Targeted Search:** Platform-specific searches for "lung cancer [advocate/survivor/doctor/expert]" to identify accounts not captured in content sampling.

**2.3 Community Nominations:** Input from patient advisory groups and advocacy organizations to identify influential voices known within the community.

# **3. Eligibility Criteria**

**Include:** Individuals or organizations with public accounts, at least 2 lung cancer-related posts in the audit period, and English-language content.

**Exclude:** Private accounts, inactive accounts (no posts in 6 months), bots or automated accounts, accounts primarily promoting unproven treatments.

# **4. Scoring Criteria (0-9 points)**

## ***4.1 Credibility (0-2 points)***

Assesses verified status, credentials, and organizational affiliation [3].

**2 points:** Platform-verified account OR licensed healthcare professional (MD, DO, NP, PA, RN) OR recognized cancer organization

**1 point:** Stated professional credentials (researcher, public health professional) OR affiliated with healthcare/academic institution

**0 points:** No verifiable credentials or organizational affiliation

## ***4.2 Reach (0-2 points)***

Platform-specific follower/subscriber thresholds reflecting audience size [4].

| **Platform** | **2 Points** | **1 Point** | **0 Points** |
| --- | --- | --- | --- |
| YouTube | ≥10,000 subscribers | 5,000-9,999 | <5,000 |
| X/Twitter | ≥25,000 followers | 10,000-24,999 | <10,000 |
| TikTok | ≥50,000 followers | 20,000-49,999 | <20,000 |
| Instagram | ≥25,000 followers | 10,000-24,999 | <10,000 |
| Facebook | ≥10,000 followers | 5,000-9,999 | <5,000 |
| Reddit | ≥50,000 karma | 10,000-49,999 | <10,000 |
| Bluesky | ≥5,000 followers | 1,000-4,999 | <1,000 |

## ***4.3 Engagement Rate (0-2 points)***

Average engagement rate across recent posts (likes + comments / followers × 100) [5].

**2 points:** ≥10% engagement rate

**1 point:** 5-9% engagement rate

**0 points:** <5% engagement rate

## ***4.4 Relevance (0-2 points)***

Frequency and recency of lung cancer-related content.

**2 points:** ≥5 lung cancer-focused posts in audit period (6-12 months)

**1 point:** 2-4 lung cancer-focused posts in audit period

**0 points:** <2 lung cancer-focused posts

## ***4.5 Multi-Platform Presence (0-1 point)***

Active presence across multiple platforms extends reach and influence.

**1 point:** Active on 2+ platforms with lung cancer content

**0 points:** Active on only 1 platform

# **5. Tier Assignment**

| **Tier** | **Score Range** | **Priority Level** | **Recommended Action** |
| --- | --- | --- | --- |
| Tier 1 | 7-9 points | High | Prioritize for partnership outreach; potential co-creation collaborators |
| Tier 2 | 4-6 points | Moderate | Include in dissemination network; potential amplification partners |
| Tier 3 | 0-3 points | Low | Monitor for growth; no active outreach at this time |

# **6. Worked Examples (*fabricated for illustrative purposes*)**

## ***Example 1: Verified Oncologist***

Dr. Jane Smith, MD - Thoracic oncologist at academic medical center, verified on X/Twitter with 35,000 followers, 12% engagement rate, 8 lung cancer posts in audit period, also active on Instagram.

• Credibility: 2 (verified + MD)

• Reach: 2 (35K on X/Twitter)

• Engagement: 2 (12%)

• Relevance: 2 (8 posts)

• Multi-platform: 1 (X + Instagram)

**Total: 9 points → Tier 1**

## ***Example 2: Lung Cancer Survivor Advocate***

Maria Garcia - Stage 3 lung cancer survivor, shares journey on TikTok with 28,000 followers, 8% engagement, 6 lung cancer videos in audit period, TikTok only.

• Credibility: 0 (no professional credentials)

• Reach: 1 (28K on TikTok)

• Engagement: 1 (8%)

• Relevance: 2 (6 posts)

• Multi-platform: 0 (TikTok only)

**Total: 4 points → Tier 2**

## ***Example 3: National Advocacy Organization***

LungCAN - National lung cancer advocacy nonprofit, verified on multiple platforms, 45,000 YouTube subscribers, 6% engagement, 15 lung cancer posts in audit period, active on YouTube, Facebook, Instagram, X.

• Credibility: 2 (verified nonprofit)

• Reach: 2 (45K on YouTube)

• Engagement: 1 (6%)

• Relevance: 2 (15 posts)

• Multi-platform: 1 (4 platforms)

**Total: 8 points → Tier 1**

## ***Example 4: Health Influencer***

HealthyLiving_Mike – General wellness influencer, Instagram only, 8,000 followers, 4% engagement, 2 posts mentioning lung cancer awareness, not verified.

• Credibility: 0 (no credentials)

• Reach: 0 (8K on Instagram)

• Engagement: 0 (4%)

• Relevance: 1 (2 posts)

• Multi-platform: 0 (Instagram only)

**Total: 1 point → Tier 3**

# **7. Validation Procedures**

**Internal review:** Two team members independently score each DOL candidate. Discrepancies >1 point are discussed and resolved.

**Community validation:** Final DOL roster is reviewed by patient advisory group and community partners for face validity and to identify missing voices.

**Documentation:** All scoring decisions are documented with rationale and evidence links.

# **8. Ethical Considerations**

• DOL data is collected from public profiles only

• No direct contact without formal partnership agreement

• DOL roster is kept confidential within research team

• Any outreach follows community engagement best practices [6]

**Rubric Version:** 1.0

**Date:** December 2025

# **References**

1. Abidin C. Internet Celebrity: Understanding Fame Online. Emerald Publishing; 2018. [ISBN: 978-1787560796]

2. Freberg K, Graham K, McGaughey K, Freberg LA. Who are the social media influencers? A study of public perceptions of personality. Public Relations Review. 2011;37(1):90-92. [doi: 10.1016/j.pubrev.2010.11.001]

3. Pilgrim K, Bohnet-Joschko S. Selling health and happiness how influencers communicate on Instagram about dieting and exercise: mixed methods research. BMC Public Health. 2019;19(1):1054. [doi: 10.1186/s12889-019-7387-8] [PMID: 31391052]

4. De Veirman M, Cauberghe V, Hudders L. Marketing through Instagram influencers: the impact of number of followers and product divergence on brand attitude. International Journal of Advertising. 2017;36(5):798-828. [doi: 10.1080/02650487.2017.1348035]

5. Araujo T, Neijens P, Vliegenthart R. Getting the word out on Twitter: the role of influentials, information brokers and strong ties in building word-of-mouth for brands. International Journal of Advertising. 2017;36(3):496-513. [doi: 10.1080/02650487.2016.1173765]

6. Israel BA, Eng E, Schulz AJ, Parker EA. Methods for Community-Based Participatory Research for Health. 2nd ed. Jossey-Bass; 2012. [ISBN: 978-1118021866]

7. Rogers EM. Diffusion of Innovations. 5th ed. Free Press; 2003. [ISBN: 978-0743222099]
